# Associations between serum uric acid and the incidence of hypertension: a Chinese senior dynamic cohort study

**DOI:** 10.1186/s12967-016-0866-0

**Published:** 2016-04-30

**Authors:** Fengjiang Wei, Ning Sun, Chunyou Cai, Shuzhi Feng, Jianli Tian, Wentao Shi, Weili Xu, Yaogang Wang, Xilin Yang, Wei-Dong Li

**Affiliations:** Research Center of Basic Medical Sciences, Tianjin Medical University, 22 Qixiangtai Road, Tianjin, 300070 People’s Republic of China; Tianjin General Hospital, Tianjin Medical University, 154 Anshan Road, Tianjin, 300052 People’s Republic of China; Aging Research Center, Department of Neurobiology, Care Sciences and Society (NVS), Karolinska Institute, Stockholm, Sweden; School of Public Health, Tianjin Medical University, 22 Qixiangtai Road, Tianjin, 300070 People’s Republic of China; Department of Epidemiology and Biostatistics, School of Public Health, Tianjin Medical University, 22 Qixiangtai Road, Tianjin, 300070 People’s Republic of China

**Keywords:** Hyperuricemia, Hypertension, Dynamic cohort study, Survival analysis

## Abstract

**Background:**

The prevalence of hyperuricemia has increased dramatically during the past several decades. Studies indicating uric acid is an independent risk factor for hypertension did not sufficiently control for other known risk factors. We explored this relationship in a comprehensive Chinese senior dynamic cohort.

**Methods:**

To investigate the relationship between serum uric acid (SUA) levels and hypertension, we carried out a 6-year retrospective study (2006–2011) in a dynamic cohort with 3591 subjects free of hypertension. The first occasion of documented hypertension per subject was the index event. A Cox proportional hazards model assessed the relationship between SUA and hypertension. Kaplan–Meier survival analysis compared incidence of hypertension among individuals with each SUA quartile. Receiver operating characteristic curves were generated to obtain the area under the curve as a prediction of hypertension from SUA levels.

**Results:**

The cumulative prevalence of hypertension in our cohort was 20.7 %. The prevalence of hyperuricemia was 17.5 %. Cox regression analysis showed that, compared with the lowest SUA quartile (<4.69 mg/dl), the 4.69–5.58, 5.58–6.52, and ≥6.52 mg/dl quartiles yielded hazard ratios (95 % confidence intervals) for hypertension of 1.652 (1.265–2.156), 2.195 (1.705–2.825), and 3.058 (2.399–3.899), respectively. Cumulative incidence of hypertension was consistently higher among individuals with hyperuricemia than among those with normal SUA levels. A Kaplan–Meier survival analysis showed that hyperuricemia predicted higher incidences of hypertension in a dose-dependent manner: hypertension onset significantly differed across SUA quartiles. SUA levels were significantly and independently associated with incidence of hypertension in our cohort.

**Conclusions:**

Our results, controlling for known risk factors, suggest that SUA level is an independent risk factor for hypertension and could be a useful indicator of hypertension.

**Electronic supplementary material:**

The online version of this article (doi:10.1186/s12967-016-0866-0) contains supplementary material, which is available to authorized users.

## Background

Uric acid is the final product of purine metabolism. Serum uric acid (SUA) concentration is precisely regulated, including secretion and reabsorption in the kidneys. Hypertension is highly prevalent, affecting approximately one-third of Americans and is a leading cause of morbidity and mortality [1]. While the etiology of hypertension is unclear in many patients, uric acid has been hypothesized to activate the renin–angiotensin system, which can lead to injury to prerenal blood vessels [2].

A number of epidemiological studies have shown that increased uric acid concentrations are associated with increased risk for developing hypertension [3, 4]. Some observations in cross-sectional analyses and longitudinal studies concluded there was a hyperuricemia-hypertension link [5–9]. Although elevated SUA levels have been predictive of hypertension in longitudinal studies, the relationship between uric acid and blood pressure is confounded by numerous factors, so controversy remains. For example, although elevated uric acid levels are often associated with established traditional cardiovascular risk factors, it is not quite clear whether uric acid is the cause or a consequence of hypertension; studies indicating uric acid as an independent risk factor did not sufficiently control for other known risk factors; thus, how uric acid causes hypertension is not fully understood [10].

To decipher the relationship between SUA and hypertension while controlling for known risk factors, including sex, BMI, eGFR, and several metabolic factors, we collected a dynamic cohort comprising primarily senior citizens (the Tianjin General Hospital cohort) in the city of Tianjin, China, and performed physical exams and clinical/biochemical tests each year from 2006 to 2011.

## Methods

### Subjects

We recruited 7032 subjects for this study from 2006 to 2011. Recruited subjects were asked to participate in an annual physical examination up to 2011. At their entry examinations, a total of 3441 subjects were found to have hypertension, and during the follow-up period, 744 out of the remaining 3591 subjects developed incident hypertension. We organized two studies: (1) a cross-sectional survey using the data at the study entry; and (2) a dynamic cohort study of the subjects who did not have hypertension at study entry. Subject recruiting protocols were reviewed and approved by the Human Ethics Committee of Tianjin Medical University. Subjects gave informed consent prior to participating in this study. All experiments were performed in accordance with relevant guidelines and regulations.

All of the subjects who were randomly assigned were eligible for this study provided they met all following criteria at the baseline: (1) no evidence of hypertension (defined as systolic blood pressure of ≥140 mmHg and/or a diastolic blood pressure of ≥90 mmHg, and/or the current use of antihypertensive medication, regardless of the recorded blood pressure); (2) no evidence of left ventricular hypertrophy/myocardial infarction/heart failure; (3) no evidence of diabetes mellitus (defined as a fasting plasma glucose ≥126 mg/dl (7.0 mmol/L), or the use of antidiabetic medications); (4) no evidence of kidney disease or gout, and no use of diuretics/allopurinol to lower uric acid levels; (5) availability of serum uric acid measurement at baseline or the follow-up examinations; and (6) availability of blood pressure at baseline or follow-up examinations.

### Annual physical exams

At each exam, all subjects were measured twice for height and weight (using identical standardized anthropometric scales), and body mass index (BMI) was calculated as body weight (kg) divided by height squared (m^2^). A well-trained nurse or doctor measured resting blood pressure once using a standard mercury sphygmomanometer with the subject in the sitting position after at least 5 min of rest. All the subjects fasted overnight for at least 12 h before blood sampling. Venous blood samples were obtained from the antecubital vein for measuring SUA levels (Additional file 1: Tables S1 and S2), fasting glucose, lipid profiles (total cholesterol, triglycerides), renal function (plasma creatinine, blood urea nitrogen), liver function (total serum protein, albumin, globulin, total and direct bilirubin, and aspartate aminotransferase), white cell count, hemoglobin tests. SUA levels were measured by enzymatic methods (Chemistry Analyzer Au2700, Olympus Medical Engineering Company, Japan). Newly diagnosed diseases during the past year and identified at the physical exam were documented.

### Definitions

Hypertension was defined as a systolic blood pressure of ≥140 mmHg and/or a diastolic blood pressure of ≥90 mmHg, and/or the current use of antihypertensive medication. For calculating incidence rates, we used the first occasion of documented hypertension per subject as the index event. According to the recommended criteria for the Chinese people [11], normal weight was defined as a BMI of 18.5–23.9 kg/m^2^, overweight as a BMI of 24.0–27.9 kg/m^2^, and obesity as a BMI ≥ 28 kg/m^2^. Values of estimated glomerular filtration rate (eGFR; ml/min/1.73 m^2^) were calculated using the equation proposed by investigators in the Chronic Kidney Disease Epidemiology Collaboration. Hyperuricemia was defined as SUA ≥ 7.0 mg/dL (420 μmol/L) in males and ≥6.0 mg/dL (360 μmol/L) in females. SUA levels were categorized by quartiles as <4.69, 4.69–5.58, 5.58–6.52, and ≥6.52 mg/dl.

In this paper, the data are divided into three groups: (1) baseline data from subjects who did not have hypertension at the study entry; (2) follow-up means calculated from specified observations starting from the time of the baseline visit through the last follow-up visit or first occasion of hypertension (index event); and (3) normal-eGFR subjects with eGFR ≥ 60 ml/min/1.73 m^2^ (chronic kidney disease was defined as eGFR < 60 ml/min/1.73 m^2^) [12].

### Data analysis

The basic characteristics of the sample are described by descriptive statistics. The data for continuous variables are reported as the mean ± SD and median (interquartile range), and the data for categorical variables are reported as percentages (%). The clinical characteristics between nonhypertensive and hypertensive groups were analyzed using the Student’s *t* test for data that were normally distributed, and the Mann–Whitney U-test for non-normal distributions, The Chi squared test was used to compare differences in percentages in the dichotomous variables. A Cox proportional hazards model was used to assess the relationship between SUA and hypertension; hazard ratios and 95 % confidence intervals (CIs) for hypertension were calculated. Kaplan–Meier survival analysis was used to compare incidence of hypertension among individuals with each SUA quartile. The log rank test was used to compare the curves. Receiver operating characteristic (ROC) curves were generated to obtain the area under the curve (AUC) as a prediction of hypertension from SUA levels. Status for hypertension, myocardial infarction, stroke, type 1 and type 2 diabetes, and 45 other diseases was documented in a Filemaker Pro database. For statistical inference, a bilateral *P*-value of <0.05 was considered statistically significant. All statistical analyses were carried out using SPSS statistical software, version 17.0 (SPSS Inc., Chicago, IL, USA) for Windows.

## Results

In our dynamic cohort study, 3591 individuals were included; the mean age of the subjects was 56.83 ± 13.28 years, with 2550 males (71.0 %; mean ± SD, 59.82 ± 13.30 years of age) and 1041 females (29.0; 57.27 ± 13.02 years of age). The characteristics of the subjects at baseline and during the follow-up period are shown in Tables 1 and 2. BMI, age, most blood measures, and the prevalence of male sex and obesity increased in the hypertensive group, but eGFR and direct bilirubin levels significantly decreased. After 6 years, cumulative prevalence of hypertension was 20.7 % (22.4 % in males, 16.7 % in females; χ^2^ = 14.307, *P* < 0.001). The prevalence of hyperuricemia was 17.5 % (19.9 % in males, 11.7 % in females; χ^2^ = 33.942, *P* < 0.001). A cross-sectional survey was organized using baseline data from 3591 subjects enrolled. Logistic regression analysis identified SUA level as a factor that increased hypertension risk (Table 3). In an unadjusted model odds ratio (95 % CIs) for hypertension, compared with the lowest SUA quartile (<4.69 mg/dl), the 4.69–5.58, 5.58–6.52, and ≥6.52 mg/dl quartiles yielded odds ratios (95 % confidence intervals) for hypertension of 1.989 (1.497–2.644), 2.975 (2.267–3.905), and 4.684 (3.596–6.102), respectively. After controlling for confounders, in adjusted models (models 2 and 3) the difference remained, and risk of hypertension was higher as SUA levels increased.

**Table 1:** Characteristics of subjects [mean ± SD or *N* (%)] during baseline and follow-up periods^a^ by status at follow-up (hypertensive, nonhypertensive)

Characteristic	Hypertensive subjects (*N* = 744)		Nonhypertensive subjects (*N* = 2847)		*P*-value hypertensive versus nonhypertensive	
	Baseline	Follow-up	Baseline	Follow-up	Baseline	Follow-up
Male (%)	570 (76.61 %)		1980 (69.55 %)		0.000	0.000
Female (%)	174 (23.39 %)		867 (30.45 %)			
Age (years)	61.47 ± 11.66	63.82 ± 11.63	55.61 ± 13.41	56.64 ± 13.33	0.000	0.000
BMI (kg/m^2^)	24.85 ± 3.04	24.76 ± 2.92	24.06 ± 3.03	24.01 ± 3.00	0.000	0.000
Overweight (BMI = 24–27.9 kg/m^2^)	350 (47.0 %)	346 (46.5 %)	1115 (40.5 %)	1130 (39.7 %)	–	–
Obese (BMI ≥ 28 kg/m^2^)	105 (14.1 %)	96 (12.9 %)	268 (9.4 %)	253 (8.9 %)	–	–
TG (mg/dl)	148.85 ± 95.69	149.73 ± 82.40	131.13 ± 95.69	134.67 ± 93.05	0.000	0.000
TC (mg/dl)	188.27 ± 32.47	189.82 ± 30.54	186.34 ± 32.47	186.73 ± 30.54	0.165	0.023
HDL (mg/dl)	51.80 ± 15.46	51.03 ± 14.69	53.35 ± 15.46	53.35 ± 15.46	0.059	0.000
eGFR (ml/min/1.73 m^2^)	86.96 ± 17.03	86.62 ± 15.69	90.51 ± 18.15	91.31 ± 22.62	0.000	0.000
≥60 (ml/min/1.73 m^2^)	711 (95.6 %)	712 (95.7 %)	2727 (95.8 %)	2738 (96.2 %)	–	–
<60 (ml/min/1.73 m^2^)	33 (4.4 %)	32 (4.3 %)	120 (4.2 %)	109 (3.8 %)	–	–
SUA (mg/dl)	6.26 ± 1.38	5.65 ± 1.20	5.50 ± 1.33	5.47 ± 1.27	0.000	0.000
BUN (mg/dl)	15.41 ± 3.53	15.49 ± 2.97	14.54 ± 3.53	14.57 ± 3.36	0.000	0.000
SCR (mg/dl)	0.92 ± 0.21	0.91 ± 0.18	0.88 ± 0.18	0.88 ± 0.17	0.000	0.000
SBP (mmHg)	140	140	119.62 ± 10.97	119.60 ± 10.46	0.000	0.000
DBP (mmHg)	90	90	70.01 ± 8.83	69.59 ± 8.41	0.000	0.000
FBG (mg/dl)	92.44 ± 22.71	98.93 ± 21.26	92.08 ± 18.02	94.42 ± 16.58	0.682	0.000
TP (g/L)	75.28 ± 4.06	74.69 ± 3.51	82.38 ± 10.83	73.68 ± 3.68	0.000	0.000
GLB (g/L)	29.66 ± 3.36	29.56 ± 3.07	28.27 ± 3.42	28.15 ± 3.27	0.000	0.000
ALB (g/L)	45.62 ± 2.61	45.12 ± 2.14	45.67 ± 2.70	45.52 ± 2.51	0.641	0.000
ALT (IU/L)	28.63 ± 22.08	25.25 ± 12.29	23.26 ± 17.98	22.19 ± 11.90	0.000	0.000
TBIL (mg/dl)	0.82 ± 0.29	0.90 ± 0.29	0.82 ± 0.31	0.84 ± 0.29	0.265	0.000
DBIL mg/dl)	0.12 ± 0.07	0.17 ± 0.10	0.19 ± 0.11	0.20 ± 0.09	0.000	0.000
Hypertension incidence (2007–2011)						
2006 n/incidence (baseline/follow-up)	0/0.00 % (932/821)					
2007 n/incidence (baseline/follow-up)	155/9.15 % (1694/1567)					
2008 n/incidence (baseline/follow-up)	191/11.27 % (1695/1636)					
2009 n/incidence (baseline/follow-up)	157/8.92 % (1761/1683)					
2010 n/incidence (baseline/follow-up)	122/6.89 % (1770/1615)					
2011 n/incidence (baseline/follow-up)	119/5.73 % (2077/2077)					

*BMI* body mass index; *TG* plasma levels of triglycerides; *TC* total cholesterol; *HDL* high-density lipoprotein; *eGFR* estimated glomerular filtration rate; *SUA* serum uric acid; *BUN* blood urea nitrogen; *SCR* serum creatinine; *SBP* systolic blood pressure; *DBP* diastolic blood pressure; *FBG* fasting plasma glucose; *TP* plasma total protein; *GLB* globulin; *ALB* albumin; *ALT* alanine aminotransferase; *TBIL* total bilirubin; *DBIL* direct bilirubin

^a^Means during the follow-up period are specified observations starting from the time of the baseline visit through the last visit or incidence of hypertension

**Table 2:** Correlation analyses among serum uric acid levels and clinical characteristics during baseline and follow-up periods

Characteristic	Baseline		Follow-up	
	*R*	*P*	*r*	*P*
Age (years)	0.044	0.009	0.024	0.151
BMI (kg/m^2^)	0.340	0.000	0.362	0.000
TG (mg/dl)	0.262	0.000	0.297	0.000
TC (mg/dl)	0.023	0.278	0.013	0.509
HDL (mg/dl)	−0.232	0.000	−0.311	0.000
eGFR (ml/min/1.73 m^2^)	−0.236	0.000	−0.195	0.000
BUN (mg/dl)	0.192	0.000	0.222	0.000
SCR (mg/dl)	0.435	0.000	0.485	0.000
SBP (mmHg)	0.171	0.000	0.165	0.000
DBP (mmHg)	0.189	0.000	0.201	0.000
FBG (mg/dl)	0.023	0.162	0.046	0.006
TP (g/L)	−0.092	0.000	0.088	0.000
GLB (g/L)	0.056	0.001	0.023	0.175
ALB (g/L)	0.080	0.000	0.105	0.000
ALT (IU/L)	0.136	0.000	0.223	0.000
TBIL (mg/dl)	0.140	0.000	0.159	0.000
DBIL (mg/dl)	−0.022	0.192	0.067	0.000

*BMI* body mass index; *TG* plasma levels of triglycerides; *TC* total cholesterol; *HDL* high-density lipoprotein; *eGFR* estimated glomerular filtration rate; *SUA* serum uric acid; *BUN* blood urea nitrogen; *SCR* serum creatinine; *SBP* systolic blood pressure; *DBP* diastolic blood pressure; *FBG* fasting plasma glucose; *TP* plasma total protein; *GLB* globulin; *ALB* albumin; *ALT* alanine aminotransferase; *TBIL* total bilirubin; *DBIL* direct bilirubin

**Table 3:** Odds ratio of SUA levels for incidence hypertension in a cross sectional survey of 3591 subjects

Model	*N* (%)	Odds ratio	95 % CI	*P*-value
Model 1: unadjusted baseline values of variables				
Quartile 1	903 (25.1 %)	–	–	0.000
Quartile 2	886 (24.7 %)	1.989	1.497–2.644	0.000
Quartile 3	905 (25.2 %)	2.975	2.267–3.905	0.000
Quartile 4	897 (25.0 %)	4.684	3.596–6.102	0.000
SUA as a continuous variable (μmol/L)	3591	1.252	1.103–1.454	0.000
Model 2: model 1 adjusted for age and gender				
Quartile 1	–	–	–	0.000
Quartile 2	–	2.107	1.568–2.833	0.000
Quartile 3	–	3.432	2.558–4.604	0.000
Quartile 4	–	5.492	4.091–7.372	0.000
SUA as a continuous variable (μmol/L)	–	1.298	1.047–1.609	0.017
Model 3: model 2 further adjusted for other confounders				
Quartile 1	–	–	–	0.000
Quartile 2	–	2.141	1.534–2.987	0.000
Quartile 3	–	3.213	2.288–4.511	0.000
Quartile 4	–	5.624	3.936–8.037	0.000
SUA as a continuous variable (μmol/L)	–	1.686	1.245–2.283	0.001

Quartiles based on serum uric acid levels: 1, <4.69 mg/dl; 2, 4.69–5.58 mg/dl; 3, 5.58–6.52 mg/dl; 4, ≥6.52 mg/dl

Model 3 was further adjusted for BMI, triglycerides, eGFR, blood urea nitrogen, serum creatinine, plasma total protein, globulin, alanine aminotransferase, and direct bilirubin

*P* < 0.05 was considered statistically significant

We conduct a dynamic cohort study of the subjects who did not have hypertension at the study entry. A Cox proportional hazards model was used to assess the relationship between SUA and hypertension. At baseline, in an unadjusted model (Table 4, model 1) the hazard ratios (95 % CIs) for hypertension in the second, third, and fourth SUA quartiles, compared with the first SUA quartile, were 1.652 (95 % CI 1.265–2.156), 2.195 (95 % CI 1.705–2.825), and 3.058 (95 % CI 2.399–3.899), respectively; after adjusting for age and sex (model 2), they were 1.644 (95 % CI 1.253–2.158), 2.274 (95 % CI 1.746–2.963), and 3.174 (95 % CI 2.448–4.114), respectively. The association was also significant after further adjustment for multiple metabolic parameters (model 3): the hazard ratios comparing the second, third, and fourth SUA quartiles versus first SUA quartile were 1.745 (95 % CI 1.321–2.328), 2.349 (95 % CI 1.774–3.111), and 3.152 (95 % CI 2.371–4.191), respectively (*P* < 0.001). When the analyses were repeated using SUA levels as a continuous variable, significant associations were observed in models 1–3 between increased SUA levels (1 μmol/L) and the risk of hypertension.

**Table 4:** Hazard ratios of SUA levels for incidence hypertension during 6 years of follow-up among 3591subjects without hypertension at the entyry examination

Model	*N* (%)	Hazard ratio	95 % CI	*P*-value
Baseline values: models 1–3				
Model 1: unadjusted baseline values of variables				
Quartile 1	903 (25.1 %)	–	–	0.000
Quartile 2	886 (24.7 %)	1.652	1.265–2.156	0.000
Quartile 3	905 (25.2 %)	2.195	1.705–2.825	0.000
Quartile 4	897 (25.0 %)	3.058	2.399–3.899	0.000
SUA as a continuous variable (μmol/L)	3591	2.039	1.747–2.379	0.000
Model 2: model 1 adjusted for age and gender				
Quartile 1	–	–	–	0.000
Quartile 2	–	1.644	1.253–2.158	0.000
Quartile 3	–	2.274	1.746–2.963	0.000
Quartile 4	–	3.174	2.448–4.114	0.000
SUA as a continuous variable (μmol/L)	–	1.703	1.292–2.243	0.000
Model 3: model 2 further adjusted for other confounders				
Quartile 1	–	–	–	0.000
Quartile 2	–	1.754	1.321–2.328	0.000
Quartile 3	–	2.349	1.774–3.111	0.000
Quartile 4	–	3.152	2.371–4.191	0.000
SUA as a continuous variable (μmol/L)	–	1.793	1.512–2.126	0.000
Follow-up values: models 4–6				
Model 4: unadjusted follow-up values of variables				
Quartile 1	897 (25.0 %)	–	–	0.018
Quartile 2	878 (24.5 %)	1.279	1.029–1.590	0.027
Quartile 3	917(25.5 %)	1.160	0.886–1.519	0.281
Quartile 4	899(25.0 %)	1.366	1.120–1.666	0.002*
SUA as a continuous variable (μmol/L)	3591	1.104	1.030–1.184	0.005*
Model 5: model 4 adjusted for age and sex				
Quartile 1	–	–	–	0.009*
Quartile 2	–	1.243	1.060–1.639	0.013*
Quartile 3	–	1.410	0.947–1.630	0.117
Quartile 4	–	1.264	1.155–1.722	0.001*
SUA as a continuous variable (μmol/L)	–	1.264	1.198–1.335	0.000
Model 6: model 5 further adjusted for other confounders				
Quartile 1	–	–	–	0.026*
Quartile 2	–	1.103	1.031–1.179	0.004*
Quartile 3	–	1.043	0.778–1.398	0.780
Quartile 4	–	1.350	1.106–1.648	0.003*
SUA as a continuous variable (μmol/L)	–	1.157	1.066–1.255	0.001*

Model 1–3 (Baseline) Quartiles based on serum uric acid levels: 1, <4.69 mg/dl; 2, 4.69–5.58 mg/dl; 3, 5.58–6.52 mg/dl; 4, ≥6.52 mg/dl

Model 4–6 (Means during follow-up period) Quartiles based on serum uric acid levels: 1, <4.64 mg/dl; 2, 4.64–5.41 mg/dl; 3, 5.41–5.78 mg/dl; 4, ≥5.78 mg/dl

Model 3 was further adjusted for BMI, triglycerides, eGFR, blood urea nitrogen, serum creatinine, plasma total protein, globulin, alanine aminotransferase, and direct bilirubin

Model 6 was further adjusted for BMI, triglycerides, total cholesterol, high-density lipoproteins, estimated glomerular filtration rate, blood urea nitrogen, serum creatinine, fasting plasma glucose, plasma total protein, globulin, albumin, alanine aminotransferase, total bilirubin, and direct bilirubin

* *P* < 0.05 was considered statistically significant

When we used means for subject characteristics during the follow-up period, in unadjusted models (Table 4, model 4), significant differences were identified in the second and fourth SUA quartile, but not the third quartile, compared with the first. After controlling for confounders, in adjusted models (models 5 and 6) the difference remained, and risk of hypertension was higher in the second and fourth SUA quartiles.

To investigate effects of impaired renal function on uric acid phenotype correlations, we divided our cohort by normal (≥60 ml/min/1.73 m^2^) and abnormal (<60 ml/min/1.73 m^2^) eGFR. The Cox regression results in normal-eGFR subjects were relatively unchanged (Table 5). In an unadjusted model (model A), the hazard ratios (95 % CIs) for hypertension in the second, third, and fourth SUA quartiles, compared with the first SUA quartile, were 1.738 (95 % CI 1.321–2.287), 2.166 (95 % CI 1.666–2.815), and 3.065 (95 % CI 2.386–3.937), respectively. After adjusting for age and sex (model B), they were 1.761 (95 % CI 1.331–2.329), 2.305 (95 % CI 1.751–3.035), and 3.320 (95 % CI 2.535–4.347), respectively. After further adjustment for multiple metabolic parameters (model C), they were 1.662 (95 % CI 1.261–2.190), 2.028 (95 % CI 1.552–2.649), and 2.747 (95 % CI 2.115–3.566), respectively (*P* < 0.001).

**Table 5:** Incidence of hypertension by serum uric acid quartile in normal-eGFR subjects

Model	*N* (%)	Hazard ratio	95 % CI	*P*-value
Model A: unadjusted				
Quartile 1	844 (24.5 %)	–	–	0.000
Quartile 2	872 (25.4 %)	1.738	1.321–2.287	0.000
Quartile 3	855 (24.9 %)	2.166	1.666–2.815	0.000
Quartile 4	867 (25.2 %)	3.065	2.386–3.937	0.000
SUA as a continuous variable (μmol/L)	3438	2.061	1.758–2.416	0.000
Model B: model A adjusted for age and sex				
Quartile 1	–	–	–	0.000
Quartile 2	–	1.761	1.331–2.329	0.000
Quartile 3	–	2.305	1.751–3.035	0.000
Quartile 4	–	3.320	2.535–4.347	0.000
SUA as a continuous variable (μmol/L)	–	2.040	1.739–2.392	0.000
Model C: model B further adjusted for other confounders				
Quartile 1	–	–	–	0.000
Quartile 2	–	1.662	1.261–2.190	0.000
Quartile 3	–	2.028	1.552–2.649	0.000
Quartile 4	–	2.747	2.115–3.566	0.000
SUA as a continuous variable (μmol/L)	–	1.807	1.519–2.150	0.000

Model C was further adjusted for BMI, TG, eGFR, blood urea nitrogen, serum creatinine, plasma total protein, globulin, alanine aminotransferase, and direct bilirubin

Quartiles based on serum uric acid levels: 1, <4.66 mg/dl; 2, 4.66–5.53 mg/dl; 3, 5.53–6.46 mg/dl; 4, ≥6.46 mg/dl

**Fig. 1:**
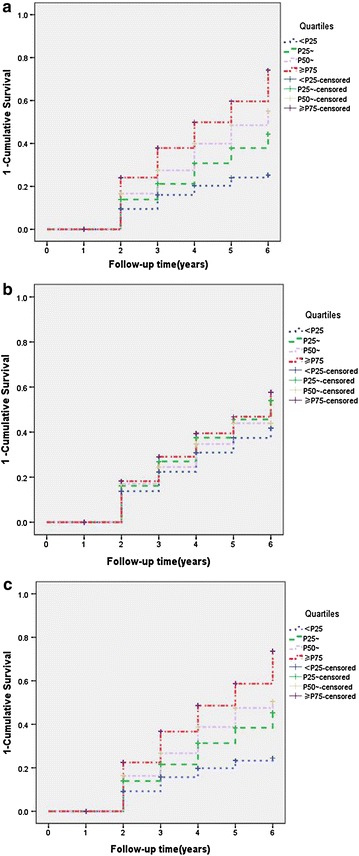
Kaplan-Meier curves for hypertension among quartiles of serum uric acid (SUA) levels. Baseline quartiles of SUA were defined as follows: first quartile, <4.69 mg/dl (<P_25_); second quartile, 4.69–5.58 mg/dl (P_25_~); third quartile, 5.58–6.52 mg/dl (P_50_~); and fourth quartile, ≥6.52 mg/dl (≥P_75_). **a** Baseline, all subjects (log-rank test *P*-value <0.01, <P_25_ vs. other quartiles). **b** Follow-up, all subjects (log-rank test *P*-value <0.05, <P_25_ vs. other quartiles). **c** Baseline, normal-eGFR subjects only (log-rank test *P*-value <0.01, <*P*_*25*_ vs. other quartiles)

Survival analyses for hypertension status in uric acid quartiles during the 6-year period (2006–2011) showed significant dose effects in each quartile. A Kaplan–Meier survival analysis showed that hyperuricemia predicted higher incidences of hypertension in a dose-dependent manner: hypertension onsets significantly differed across SUA quartiles (Fig. 1a, b).

Because SUA level is considered as an index of renal function, we divided subjects by eGFR and reexamined the correlations. We found that most correlations between SUA and hypertension and liver function phenotypes remained essentially unchanged in the normal-eGFR group (Table 5; Fig. 1c).

**Fig. 2:**
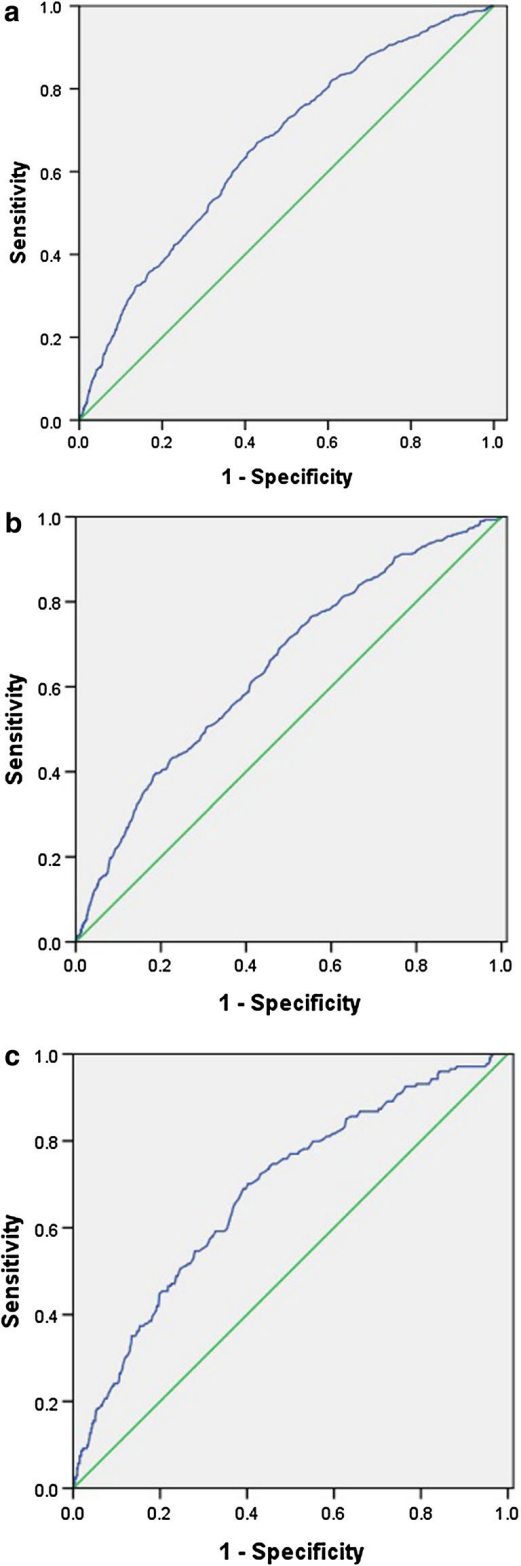
Receiver operating characteristic curve for the prediction of hypertension from serum uric acid levels in total data [**a**; area under the curve (AUC) = 0.659], in men (**b**; AUC = 0.647), and in women (**c**; AUC = 0.683)

Receiver operating characteristic curves were generated to obtain the AUC as a prediction of hypertension from SUA levels. ROC analysis revealed an AUC of 0.659 in the ability of SUA levels to predict hypertension diagnosis in the total data. This model was also found to be significantly different than the random predictor (AUC = 0.500, *P* < 0.001). The sensitivity and specificity were 65.1 and 58.8 %, respectively. For men, the sensitivity and specificity were 71.6 and 49.7 %, respectively, and the AUC was 0.647. For women, the sensitivity and specificity were 70.1 and 59.8 %, respectively, and the AUC was 0.683 (Fig. 2a–c).

## Discussion

In a Chinese senior dynamic cohort (the Tianjin General Hospital cohort), we found a significant association between higher SUA concentrations and an increased hazard of incident hypertension. This association was independent of age, sex, BMI, triglycerides, eGFR, blood urea nitrogen, serum creatinine, plasma total protein, globulin, alanine aminotransferase, and direct bilirubin. Using SUA measurements as a continuous measure or a dichotomous variable did not change our findings. There were consistent findings across the various adjusted and unadjusted analyses. These results demonstrate that SUA level is a durable marker of risk for hypertension. The relative risk of hypertension increased in a dose-dependent manner with increasing uric acid quartiles.

Our findings relating SUA to hypertension incidence with short-term follow-up (up to 5 years) confirm several previous reports. Our results are similar to observations in cross-sectional analyses and longitudinal studies [5–7], and several studies report linear hazard ratios comparable to ours [8–14]. In the Multiple Risk Factor Intervention Trial (MRFIT) study [8] and the Beaver Dam study [9] the hazard ratios for hypertension were 1.02 and 1.31, respectively. Our reported hazard ratios are larger than those in the MRFIT study but smaller than in the Beaver Dam study. In our cohort, the cumulative prevalence of hypertension was 20.7 % (22.4 % in men, 16.7 % in women). Zhang et al. reported prevalences of hypertension as 19.0 % in men and 11.0 % in women in a Chinese community [14], and Sundström et al. [15] reported a hypertension prevalence of 13.8 %. Compared with these previous studies, our cohort had a high cumulative incidence of hypertension, and the cause of this difference might be our older study population. The prevalence of hypertension was somewhat different from the general population in China. Qi et al. [16] reported that the adjusted prevalence of hypertension was 20.9 %, which is very close to ours, but Yang et al. [17] reported a higher prevalence of 30.8 % in northeastern China by a cross-sectional study.

The survival analysis showed that hyperuricemia predicted higher incidences of hypertension in a dose-dependent manner: hypertension onset significantly differed across SUA quartiles, a result consistent with that of Masuo et al. [18]. Despite higher hazard ratios in the top quartile for SUA, in our dynamic cohort the SUA levels dropped across all quartiles over the follow-up period. These results are somewhat contradictory to previous longitudinal studies [9, 10]. The disparity may be due to three main reasons. First, in the present study, we did not assess lifestyle-related variables such as smoking, alcohol, physical training, and eating habits, which may have affected SUA levels during the follow-up period. Second, there is known variation in SUA level when measured repeatedly, and because several drugs that may also have affected SUA levels were not assessed, we could not pursue this question. The last reason may result from our dynamic cohort: as new, younger subjects joined the cohort and older subjects were lost to follow-up, our results may be partially due to the younger age of more recent subjects.

Our study had strengths and limitations. A strength of our study is that the cross-sectional study and the dynamic cohort study generated consistent results. However, one limitation was that we did not collect data on lifestyle factors and socioeconomic status, which are known to vary in risk of hypertension [19, 20]. Despite this, the association between SUA and hypertension is unlikely mediated by these factors, and undiagnosed high SUA is less likely to cause changes in lifestyle and socioeconomic status. Another limitation was that the subjects were “healthy persons” who had routine health examinations but were not patients who sought medical care. The hospital was a top tertiary care center, and persons who had annual health examinations at this hospital were likely to civil servants, university teachers, and employees of government-funded agencies—persons of high socioeconomic status. Thus, they are not representative of the general population. Further replication our findings in general population is warranted, especially among subjects with low socioeconomic status.

## Conclusions

We found that SUA levels predicted incident hypertension in a Chinese cohort of senior persons. Increased SUA levels were significantly and independently associated with the incidence of hypertension over a 6-year period in a dynamic cohort without hypertension at baseline. A survival analysis showed that elevated SUA levels predicted higher cumulative incidence of hypertension. To prevent cardiovascular events, this relationship between increased SUA levels and increased incidence of hypertension should be noted. In future research, we plan to investigate clinical outcomes of individuals with elevated SUA levels in our cohort and test genetic backgrounds related to hyperuricemia in general populations.

## Additional file

10.1186/s12967-016-0866-0 Supplement Tables S1 and S2.

## Abbreviations

SUA: serum uric acid
BMI: body mass index
eGFR: estimated glomerular filtration rate
CI: confidence interval
SD: standard deviation
ROC: receiver operating characteristic
AUC: area under the curve
MRFIT: multiple risk factor intervention trial
