# Supplementary material for: Associations between serum uric acid and the incidence of hypertension: a Chinese senior dynamic cohort study
Source: J Transl Med. 2016 Apr 30;14:110. doi: 10.1186/s12967-016-0866-0 (PMC4851787; doi:10.1186/s12967-016-0866-0)
Supplement: Supplementary file 1 — 10.1186/s12967-016-0866-0 Supplement Tables S1 and S2. [file 12967_2016_866_MOESM1_ESM.docx]

**Table S1 Characteristics of subjects [mean±SD or *N* (%)] during baseline by SUA quartiles**

| Characteristic | Quartile 1 | Quartile 2 | Quartile 3 | Quartile 4 |
| --- | --- | --- | --- | --- |
| Male(%) | 345(38%) | 607(69%) | 767(85%) | 831(93%) |
| Female(%) | 558(62%) | 279(31%) | 138(15%) | 66( 7%) |
| Age (years) | 55.77±13.99 | 57.13±13.21 | 56.79±12.57 | 58.71±13.25 |
| BMI (kg/m^2^) | 22.73± 2.89 | 23.93± 2.93 | 24.68± 2.80 | 25.56± 2.84 |
| TG (mg/dl) | 104.55± 67.34 | 128.47±88.60 | 140.00±85.95 | 167.45± 97.46 |
| TC (mg/dl) | 186.34±34.79 | 185.95±31.31 | 188.27±32.09 | 187.50±32.47 |
| HDL (mg/dl) | 54.51±21.26 | 53.74±12.37 | 51.42±11.21 | 50.26±10.44 |
| eGFR (ml/min per 1.73 m^2^) | 95.17±18.28 | 91.32±16.81 | 88.73±17.36 | 83.92±17.55 |
| SUA (mg/dl) | 4.00±0.53 | 5.14±0.25 | 6.01±0.27 | 7.47±0.86 |
| BUN (mg/dl) | 13.89±3.31 | 14.18±3.31 | 14.79±3.45 | 15.69±3.84 |
| SCR (mg/dl) | 0.77±0.15 | 0.86±0.16 | 0.92±0.17 | 0.99±0.20 |
| SBP (mmHg) | 117.94±11.89 | 120.35±10.88 | 122.60±10.74 | 124.28±10.04 |
| DBP (mmHg) | 67.93± 8.81 | 70.13± 8.79 | 71.87± 8.60 | 73.39± 8.70 |
| FBG (mg/dl) | 91.54±25.23 | 92.44±19.28 | 92.08±19.28 | 92.62±17.12 |
| TP (g/L) | 82.40±10.82 | 81.33±10.58 | 79.78± 9.78 | 80.15± 9.52 |
| GLB (g/L) | 28.52± 3.54 | 28.31± 3.41 | 28.59± 3.31 | 28.83± 3.53 |
| ALB (g/L) | 45.39± 2.71 | 45.59± 2.69 | 45.65± 2.63 | 46.00± 2.68 |
| ALT (IU/L) | 21.59±30.68 | 23.16±11.27 | 25.19±14.34 | 27.55±13.07 |
| TBIL (mg/dl) | 0.77±0.29 | 0.81±0.30 | 0.85±0.32 | 0.87±0.29 |
| DBIL (mg/dl) | 0.18±0.11 | 0.17±0.10 | 0.17±0.10 | 0.17±0.10 |

**Table S2 Characteristics of subjects [mean±SD or *N* (%)] during follow-up by SUA quartiles**

| Characteristic | Quartile 1 | Quartile 2 | Quartile 3 | Quartile 4 |
| --- | --- | --- | --- | --- |
| Male(%) | 314(35%) | 610(69%) | 785(86%) | 841(94%) |
| Female(%) | 583(65%) | 268(31%) | 132(14%) | 58( 6%) |
| Age (years) | 57.10±13.97 | 58.89±13.21 | 58.75±12.67 | 59.20±13.10 |
| BMI (kg/m^2^) | 22.65± 2.82 | 23.89± 2.81 | 24.27± 2.86 | 25.27± 2.80 |
| TG (mg/dl) | 104.55±57.59 | 129.36±78.85 | 136.44±68.22 | 164.80±110.75 |
| TC (mg/dl) | 186.73±31.31 | 187.89±30.93 | 188.27±29.00 | 187.50±30.54 |
| HDL (mg/dl) | 54.90±20.88 | 54.12±11.98 | 51.80±8.51 | 50.64±7.35 |
| eGFR (ml/min per 1.73 m^2^) | 95.28±17.89 | 92.41±29.69 | 89.65±15.11 | 86.08±17.69 |
| SUA (mg/dl) | 3.98±0.50 | 5.04±0.23 | 5.60±0.11 | 6.74±0.82 |
| BUN (mg/dl) | 13.78±3.05 | 14.62±3.05 | 15.01±3.14 | 15.41±3.50 |
| SCR (mg/dl) | 0.77±0.14 | 0.86±0.15 | 0.91±0.13 | 0.97±0.18 |
| SBP (mmHg) | 119.55±12.24 | 122.08±12.45 | 123.57±11.20 | 124.55±10.51 |
| DBP (mmHg) | 68.03± 8.58 | 69.89± 8.49 | 71.88± 8.45 | 72.42± 8.43 |
| FBG (mg/dl) | 94.61±21.44 | 95.51±18.38 | 93.88±14.42 | 96.05±15.68 |
| TP (g/L) | 73.68± 3.86 | 73.59± 3.52 | 73.39± 3.54 | 74.35± 3.63 |
| GLB (g/L) | 28.49± 3.43 | 28.29± 3.14 | 28.02± 2.94 | 28.63± 3.34 |
| ALB (g/L) | 45.18± 2.46 | 45.28± 2.41 | 45.36± 2.49 | 45.72± 2.41 |
| ALT (IU/L) | 19.44±10.50 | 22.02±12.81 | 22.93±10.74 | 25.42±12.22 |
| TBIL (mg/dl) | 0.79±0.27 | 0.84±0.27 | 0.87±0.27 | 0.90±0.31 |
| DBIL (mg/dl) | 0.19±0.09 | 0.19±0.08 | 0.19±0.08 | 0.20±0.11 |
